# Assessing the Effects of the New Cooperative Medical Scheme on Alleviating the Health Payment-Induced Poverty in Shaanxi Province, China

**DOI:** 10.1371/journal.pone.0157918

**Published:** 2016-07-05

**Authors:** Xiaowei Yang, Jianmin Gao, Zhongliang Zhou, Jue Yan, Sha Lai, Yongjian Xu, Gang Chen

**Affiliations:** 1 School of Public Health, Health Science Center, Xi’an Jiaotong University, Xi’an, Shaanxi, People’s Republic of China; 2 School of Public Policy and Administration, Xi’an Jiaotong University, Xi’an, Shaanxi, People’s Republic of China; 3 School of Medicine, Flinders University, Adelaide, Australia; Old Dominion University, UNITED STATES

## Abstract

**Background:**

Disease has become one of the key causes of falling into poverty in rural China. The poor households are even more likely to suffer. The New Cooperative Medical Scheme (NCMS) has been implemented to provide rural residents financial protection against health risks. This study aims to assess the effect of the NCMS on alleviating health payment-induced poverty in the Shaanxi Province of China.

**Methods:**

The data was drawn from the 5^th^ National Health Service Survey of Shaanxi Province, conducted in 2013. In total, 41,037 individuals covered by NCMS were selected. Poverty headcount ratio (HCR), poverty gap and mean positive poverty gap were used for measuring the incidence, depth and intensity of poverty, respectively. The differences on poverty measures pre- and post- insurance reimbursement indicate the effectiveness of alleviating health payment-induced poverty under NCMS.

**Results:**

For the general insured, 5.81% of households fell below the national poverty line owing to the health payment; this HCR dropped to 4.84% after insurance reimbursement. The poverty HCRs for the insured that had hospitalization in the past year dropped from 7.50% to 2.09% after reimbursement. With the NCMS compensation, the poverty gap declined from 42.90 Yuan to 34.49 Yuan (19.60% decreased) for the general insured and from 57.48 Yuan to 10.01 Yuan (82.59% decreased) for the hospital admission insured. The mean positive poverty gap declined 3.56% and 37.40% for two samples, respectively.

**Conclusion:**

The NCMS could alleviate the health payment-induced poverty. The effectiveness of alleviating health payment-induced poverty is greater for hospital admission insured than for general insured, mainly because NCMS compensates for serious diseases. Our study suggests that a more comprehensive insurance benefit package design could further improve the effectiveness of poverty alleviation.

## Introduction

Illness and health care use may change the economic status of households with the impact of direct costs from medical treatment and related financial costs, productive time losses resulting from illness and other household adoption of illness. There is some evidence that proves health payments could induce poverty and thus households have more illness [1–2]. The World Bank, the World Health Organization (WHO) and other non-governmental organizations have devoted investment in health to reduce poverty [3–4]. As health is one of the basic human rights, no one ought to be pushed into poverty, or further into poverty because of health payments [5].

In China, disease has become the main reason that causes households to fall into poverty, and others already in poverty to sink deeper. In 2008, there were 9.1% households officially identified as poor by the local government, and 34.5% of them declared disease and injury to be the main reason for their poverty. In rural China, it was more severe, with 37.8% of poor households attributing disease and injury to poverty [6]. Establishing universal health insurance coverage is one tool that government can adopt to address uncertainty in health care. One of its greatest merits is that health insurance may help reduce the financial burden faced by patients in accessing health services, which in turn increases access to health services. Since 2000, many developing countries in the world have sought to establish universal health insurance schemes for their nations [7].

The New Cooperative Medical Scheme (NCMS) is a voluntary basic medical insurance that was piloted nationwide by the State government in 2003 to protect the 750 million rural populations from medical impoverishment. Initially, there were three counties piloting the NCMS in Shaanxi Province, the province where this study was conducted. By the end of 2013, NCMS has covered 25.5 million people, about 99.4% of the total rural population of Shaanxi Province [8]. In 2013, per capital financing for NCMS amounted to 340 Yuan (US$ 54.84, average exchange rate 1 US$ = 6.20 Yuan reported by the World Bank in 2013), of which 60 Yuan (17.6%) was paid by individuals, the remaining was subsidized by the government [9]. The detailed benefit package of NCMS of Shaanxi Province in 2013 is summarized in Table 1[10]. From Table 1 we can see that the NCMS mainly compensated hospitalization expenses in Shaanxi Province.

**Table 1:** Benefit package of NCMS in Shaanxi Province in 2013.

Hospital level	Outpatient	Chronic or fatal disease	Inpatient	
	Compensation rates (%)	Compensation rates (%)	Deductible (Yuan)	Compensation rates (%)
Village	75	60	-	-
Township	65	60	150	90
County	-	60	400	80
City secondary	-	60	500	75
City tertiary	-	60	1000	60
Provincial secondary	-	60	2000	65
Provincial tertiary	-	60	3000	55
Celling(Yuan)	Number of people insured within a household times 100	20,000 for one insured person, including 20,000 for fatal disease or 5,000 for chronic disease.	130,000	

Note: the NCMS classified chronic diseases into two groups: the first group including uremia, renal dialysis, cancer chemotherapy, leukemia, and the second group including diabetes, hypertension, et al. Celling lines were 20,000 Yuan for the first group and 5,000 Yuan for the second group.

The financial protection effects of the NCMS on the insured rural residents in China have been reported in the literature. Using the data collected from three pilot counties in Hubei Province in 2004, one year after the pilot of the NCMS, Chen et al. found that the head-count index increased from 4.03% to 24.19% after hospitalization expenditure, whilst the index dropped to 19.53% after NCMS compensation [11]. Evidence from one county in Shandong Province in 2004 showed that 5.06% of the sample households fell below the national poverty line due to health payments, compared with 4.03% after reimbursements; in addition, with the NCMS reimbursements, the health payment-induced poverty gap dropped by 19.2% [12]. Yip and her colleagues conducted an experiment in Western and Central provinces of China in 2007, with a benefit package design consisting of an individual medical saving account for outpatient services and a pooled account for inpatient services. Their results suggest that under the international poverty line of US$1.08 per person per day, the insurance reduced the poverty headcount by 3.5–3.9% and the average poverty gap by 11.8–16.4% [13]. So far there is one study analyzing the financial protection effect of the NCMS in Shaanxi Province [14], using data from a pilot county in 2007. It was found that the NCMS had reduced the poverty head-count from 18.85% to 13.93%, whilst the poverty gap index decreased from 19,280 Yuan to 12,221 Yuan, the income gap index decreased from 96.46% to 82.73%. The studies above have demonstrated that the NCMS plays an important role in alleviating health payment-induced poverty. However, previous studies were only based on data from pilot counties when the NCMS had just been implemented. The insured respondents would likely be self-selected into the insurance scheme.

The purpose of this study is to assess the financial protection effect on alleviating health payment-induced poverty under NCMS in Shaanxi Province. Differing from previous studies, this study was conducted using provincial representative data under the context of almost universal health insurance coverage in rural areas. The finding of this study will provide readers with information about the effectiveness of NCMS implementation, and sheds light on relevant policy development to optimize NCMS.

## Method

### Data

The data comes from the 5^th^ National Health Services Survey (NHSS) of Shaanxi Province. The NHSS was organized and implemented by Shaanxi Health and Family Planning Commission (HFPC). For each household, a face-to-face interview was conducted by a trained investigator using a hard-copy questionnaire. Shaanxi Province is located in the northwest of China, covering an area of 205,800 Km^2^. In 2013 it had a population of 37.6 million, among them 48.69% lived in rural areas. The per capita Gross Domestic Product of Shaanxi Province was 42,690 Yuan (US$ 6,893, based on an exchange rate of 1 US$ = 6.1932 Yuan), ranking 13^th^ of all the provinces in China [15].

A four-stage stratified random sampling was used to collect a representative sample of Shaanxi Province. This sampling frame was designed by Shaanxi HFPC. In brief, 32 counties (districts) were randomly selected in the first stage, among which 160 towns were sampled in the second stage. Two villages were sampled in each town in the third stage, and 60 households were sampled in each village at the fourth stage. In total, there were 20,700 households (57,529 people) sampled in the 5^th^ NHSS of Shaanxi Province. The Myer’s Index of this sample is 1.65, which indicates superior representation of the whole population. This paper only used the data of NCMS insured, consisting of a sample size of 41,037 respondents.

### Health payment-induced poverty

Health payment-induced poverty refers to the poverty absolutely attributable to health payments. It is measured by the difference between the poverty before health payments are included in total household consumption expenditure, and poverty after they are subtracted [12] (net of health payment). Households may adopt many changes to cope with impoverishing health payments [16], which may influence household income or expenditure. The health payment-induced poverty measurement assumes that household expenditure is not responsive to the health payments and variation of expenditure is ignored. Thus, the ranking in the consumption expenditure distribution of households is fixed.

In the NHSS, respondents reported the total hospitalization cost, as well as the out-of-pocket (OOP) hospitalization cost. By using the above information, the NCMS reimbursement on hospitalization was calculated. However, the information regarding the outpatient utilization was only limited to the OOP expense. Total outpatient cost and NCMS reimbursement were both unavailable. Thus, this study focused on the financial protection effect of the NCMS on inpatient utilization only.

Typically poverty is measured by comparing an individual’s income or consumption expenditure with a threshold, i.e. a poverty line (PL). Poverty lines are either absolute or relative. An absolute poverty line defines the cost of reaching subsistence nutritional requirements, whilst a relative poverty line indicates some fraction of mean or median household expenditure [17]. In this study, we adopted an absolute poverty line of 2300 Yuan, announced by the National Bureau of Statistic (NBS) of China in 2012. Using Mating Method, this absolute poverty line of 2300 Yuan was calculated based on the food consumption.

### Economic status

Household’s economic status could be potentially denoted by annual household income or annual household consumption expenditure in the NHSS data. One family respondent, who is more familiar with the household financial situation, was asked to report the total household income and consumption expenditure for the past year. In the NHSS questionnaire, household consumption expenditure was categorized into eight components: food, daily living, transportation and communication, housing, education, entertainment, health care, and others. Although both measures were self-reported, the literature suggested that self-reported income is subjected to larger reporting bias than the consumption expenditure information, i.e. respondents in developing countries tended to under-report true income in the survey [18]. As such, following the literature, we opted to use the household consumption expenditure to proxy economic status in this study.

### Family scale adjustment

To calculate the per capita annual consumption expenditure, the family scale needs to be adjusted. Simply using the household size as a denominator may not be ideal, since children and adults have different needs. In this study, the household scales were adjusted as the number of adult equivalents using Eq 1[19]:

$$\text{AE}={\left(\text{A}+\alpha \text{K}\right)}^{\theta },$$ (1)

Where *AE* refers to adult equivalent, *A* refers to number of adults, *K* refers to number of children. α is the “cost of children” equaling to 0.3 in developing countries. θ refers to the scale of family economy, and set to be 0.75 in developing countries[20].

### Poverty measures

Three poverty measures were investigated, including poverty incidence, poverty depth and poverty intensity [21].

Let x_i_ be the per capita annual expenditure of household i. Poverty headcount ratio (HCR) shows the proportion of the population that lives below the poverty line. The HCR is calculated based on Eq 2:

$$H=\frac{\sum_{i=1}^{N}s_{i}p_{i}}{\sum_{i=1}^{N}s_{i}},$$ (2)

Where p_*i*_ = 1 if *x*_*i*_<PL and is 0 otherwise, *s*_*i*_ is the adjusted household, and N is the number of households in the sample.

Poverty gap (G) measures the “depth” of the poverty. It is defined as the mean shortfall of the total population from the poverty line. The individual poverty gap is g_*i*_ = *p*_*i*_(*PL* − *x*_*i*_), thus the poverty gap of the whole sample could be calculated using Eq 3:

$$G=\frac{\sum_{i=1}^{N}s_{i}{\text{g}}_{i}}{\sum_{i=1}^{N}s_{i}}.$$ (3)

When making comparisons across countries with different poverty lines and currency units, a normalized poverty gap is convenient to use, which is calculated based on Eq 4:

$$NG=\frac{G}{\text{PL}}.$$ (4)

The intensity of poverty is measured using the mean positive poverty gap (MPG) (see Eq 5), which represents the average deficit of the poor from the poverty line.

MPG=GH. (5)

To assess the effect of NCMS on poverty alleviation, we firstly compare the poverty measures before and after health payment. Then we further compare the poverty indexes after the NCMS compensation.

### Ethical approval

Participants provided their verbal informed consent to participate in this study. Before the survey, Shaanxi Health and Family Planning Commission delivered a governmental document to call for cooperation from the sample counties. In order to be access to the participates smoothly, the guiders from the sample counties would contact with each participates before the survey to get their consent about the survey. If the participants agreed to accept the interview, the guider would make an appointment with them. Then the investigators went to participants’ house to do the survey.

Only the participants agreed to be interviewed, their information could be collected in the questionnaire, which means if we have the participants’ questionnaires, we have got the participants’ consent all ready.

This study was approved by the Ethics Committee of Xi’an Jiaotong University Health Science Center, and conforms to the ethics guidelines of the Declaration of Helsinki.

## Results

### Socio-demographic characteristics

Among the whole NCMS insured sample (N = 41,037), 51.23% respondents are female, 49.94% aged 45 years and older, 79.16% were married, 53.88% received middle school education or above, and 78.77% were employed. The mean per capita annual consumption expenditure and health payment were 11,602 Yuan and 2,152 Yuan, respectively. A sub-sample of respondents (N = 4,028) who had been hospitalization in the past 12 months when the NHSS survey was conducted, was also analyzed separately since the NCMS mainly reimbursed the inpatient health services. For this hospital admission insured, the per capita annual consumption expenditure was 14,570 Yuan, and per capita annual health payment was 5,681 Yuan. For detailed socio-demographic characteristics of the respondents see Table 2.

**Table 2:** Socio-demographic characteristics (percent/mean).

	General insured	Hospital admission insured
Gender		
Male	48.77	41.56
Female	51.23	58.44
Age groups		
0–14	13.4	9.61
15–44	36.66	25.1
45–59	28.78	28.03
≥60	21.16	37.26
Marital status		
Unmarried	12.01	3.95
Married	79.16	83.55
Widowed	7.88	11.92
Divorced	0.84	0.49
Others	0.12	0.08
Education status		
Illiterate	18.43	26.17
Primary school	27.68	32.11
Secondary school and above	53.88	41.73
Employment status		
Employed	78.77	70.61
Retirement	0.77	1.48
Student	3.76	0.93
Unemployed	16.96	26.98
Economic status		
per capita annual consumption expenditure (Yuan)	11,602 (8,182)	14,570 (9,982)
per capita annual out-of-pocket health payment (Yuan)	2,152 (3,794)	5,681 (6,932)
N	41,037	4,028

Standard deviations are in the parentheses.

### Effect of NCMS on poverty alleviation for the general insured

Table 3 shows the poverty measures firstly before and after health payments (Columns 1 & 2), and then after insurance compensation (Column 5) for the general insured. When assessing on the basis of total household consumption, 2.55 percent of the NCMS insured population in Shaanxi Province is estimated to be in poverty, according to the national poverty line (2300 Yuan). After health payments, the poverty headcount rises to 5.81 percent. This represents a sharp rise of 127.84 percent increase in poverty incidences. The estimated poverty gap also dramatically rose 166.96 percent, from 16.07 Yuan pre-health payment, to 42.90 Yuan post health payment. Expressed as a percentage of the poverty line, the poverty gap increases from 0.70 percent to 1.87 percent after health payments. The mean positive poverty gap also increases from 630.00 Yuan to 738.16 Yuan (a rise of 17.17 percent), representing a deepening of the poverty of the already poor. When expressed as a percentage of the poverty line, the normalized mean positive poverty gap increased from 27.39 percent to 32.09 percent after health payments.

**Table 3:** Measures of poverty gross and net of health payments and after compensation for all NCMS insured.

	Gross of health payments(1)	Net of health payments(2)	Difference		After compensation(5)	Difference	
			Absolute(3) = (2)-(1)	Relative(4) = [(3)/(1)]*100		Absolute(6) = (5)-(2)	Relative(7) = [(6)/(2)]*100
Poverty headcount ^**a**^	2.55% (0.0008)	5.81% (0.0012)	3.26%	127.84%	4.84% (0.0011)	-0.97	-16.70%
Poverty gap ^**b**^ (Yuan)	16.07 (0.6084)	42.90 (1.0377)	26.83	166.96%	34.49 (0.9288)	-8.41	-19.60%
Normalized poverty gap ^**b**^	0.70% (0.0003)	1.87% (0.0005)	1.17%	167.14%	1.50% (0.0004)	-0.37%	-19.79%
Mean positive poverty gap ^**c**^ (Yuan)	630.00 (14.1218)	738.16 (10.1805)	108.16	17.17%	711.86 (11.1848)	-26.30	-3.56%
Normalized mean positive poverty gap ^**c**^	27.39% (0.0061)	32.09% (0.0044)	4.70%	17.16%	30.95% (0.0049)	-1.14%	-3.55%

Standard errors presented in the parentheses.

Test the difference between the payments with and without compensation:

^**a**^ *χ*^2^ = 38.07 p<0.001

^**b**^ t = 19.45 P<0.001

^**c**^ t = 4.63 P<0.001.

People enrolled in the NCMS could get reimbursement when they fall ill and receive medical treatment. The compensation would affect the economic status of the inpatient. As is shown in Column (5) of Table 3, the poverty headcount declines to 4.84 percent after compensation. Thus, 0.97 percent of the study population is not counted as poor after insurance reimbursement. This represents a decrease of 16.70 percent in the estimated poverty. The estimated poverty gap also comes down by 19.60 percent, from 42.90 Yuan to 34.49 Yuan. Expressed as a percentage of the poverty line, the poverty gap falls off from 1.87 percent to 1.50 percent when NCMS compensates the insured. The mean positive poverty gap declines from 738.16 Yuan to 711.86 Yuan, representing a drop of 3.56 percent. This illustrates that the estimated intensity of poverty is changed slightly by NCMS compensation.

**Fig 1:**
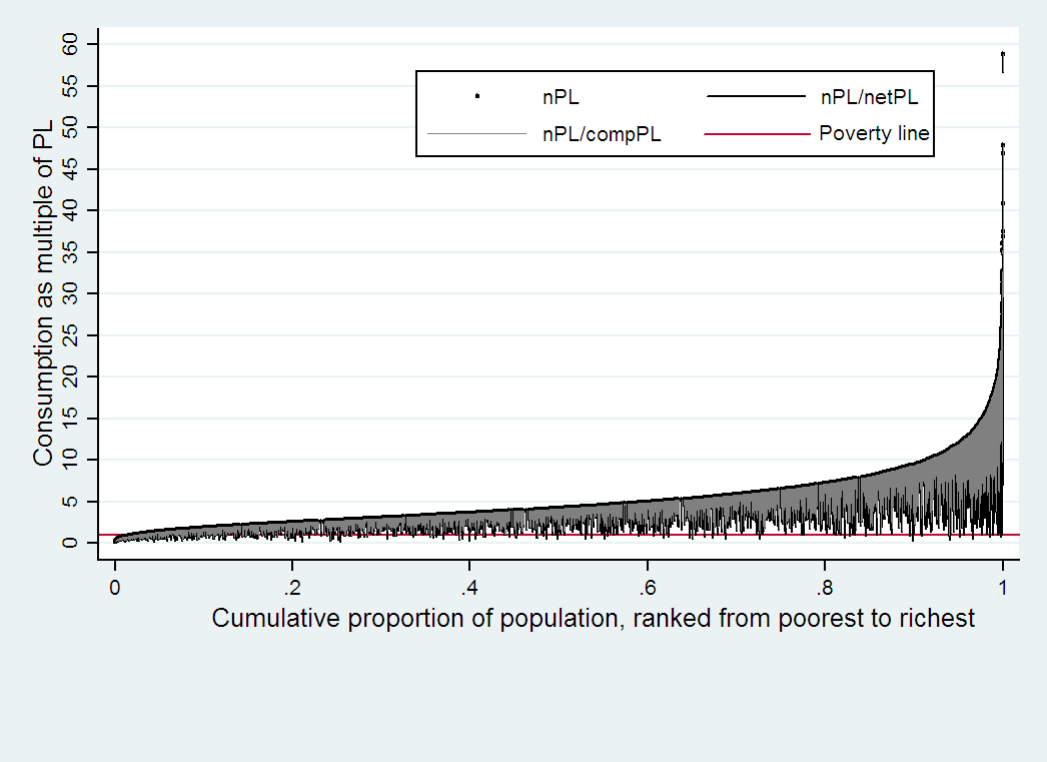
Effect of health payments and NCMS compensation on Pen’s parade of the household consumption distribution for whole NCMS insured. The black vertical not covered by the grey vertical represents the consumption reducing from NCMS compensation. When the black bar crosses the poverty line and the grey bar goes back above the poverty line, it represents the poverty alleviation by the NCMS.

The effect of health payment on poverty could be seen more clearly using the Pen’s parade (Fig 1). The horizontal axis represents the ranking in the consumption expenditure distribution, whilst the vertical axis is the per capita consumption expenditure. The horizontal red line is the poverty line. The curve represents gross household consumption with health payments, while the black vertical bar shows the extent to which the subtraction of health payments reduces consumption for each household. The bar crossing the poverty line represents a person who is not counted as poor on the basis of gross consumption, but is poor on the basis of the net consumption. The grey vertical bar shows the extent to which household consumption (net of health payments) plus compensation for everyone. Thus the black vertical not covered by the grey vertical represents the consumption reducing from NCMS compensation. When the black bar crosses the poverty line and the grey bar goes back above the poverty line, it represents the poverty alleviation by the NCMS.

As can be seen in Fig 1, health payments were larger when respondents were richer. However, the poor were more likely to fall below the poverty line after health payments. Insurance reimbursement shows financial protection effects.

### Effect of NCMS on poverty alleviation for the hospitalized insured

As introduced previously, the NCMS mainly reimburse inpatient health services. Thus, it is expected that the insurance’s financial protection effect would be stronger for those who had been hospitalized.

The results of the sub-sample analyses are presented in Table 4. As can be seen, before health payments, only 0.45 percent of the respondents are regarded to be in poverty, according to the national poverty line. There was a large rise of 1566.67 percent in the incidence of poverty (amount to 7.05 percent) after health payments. The poverty gap also acutely rose by 3222.54 percent, from 1.73 Yuan to 57.48 Yuan. The normalized poverty gap increased from 0.08 percent to 2.50 percent (representing a 3025.00 percent increase). The mean positive gap and the normalized mean positive gap both increase about 100 percent. These indexes of poverty, gross and net of health payments, illustrate that the health payments play a significant effect on household economic status for those who has been hospitalized.

**Table 4:** Measures of poverty gross and net of health payments and after compensation for hospital admission insured.

	Gross of health payments(1)	Net of health payments(2)	Difference		After compensation(5)	Difference	
			Absolute(3) = (2)-(1)	Relative(4) = [(3)/(1)]*100		Absolute(6) = (4)-(2)	Relative(7) = [(5)/(2)]*100
Poverty headcount ^**a**^	0.45% (0.0011)	7.50% (0.0042)	7.05%	1566.67%	2.09% (0.0023)	-5.41%	-72.21%
Poverty gap ^**a**^ (Yuan)	1.73 (0.5928)	57.48 (3.8075)	55.75	3222.54%	10.01 (1.3500)	-47.47	-82.59%
Normalized poverty gap ^**b**^	0.08% (0.0003)	2.50% (0.0017)	2.42%	3025.00%	0.44% (0.0006)	-2.06%	-82.47%
Mean positive poverty gap ^**b**^ (Yuan)	386.51 (99.3997)	766.60 (27.9439)	380.09	98.34%	479.92 (38.7010)	286.68	-37.40%
Normalized mean positive poverty gap ^**c**^	16.80% (0.0432)	33.33% (0.0121)	16.53%	98.39%	20.87% (0.0168)	12.46%	-37.38%

Standard errors presented in the parentheses.

Test the difference between the payments with and without compensation:

^**a**^ *χ*^2^ = 129.32 p<0.001

^**b**^ t = 14.46 P<0.001

^**c**^ t = 5.27 P<0.001.

With the NCMS compensation, all indicators of poverty measures improved. Poverty headcount and poverty gap reduced about 80 percent and the mean positive poverty gap came down 37 percent.

**Fig 2:**
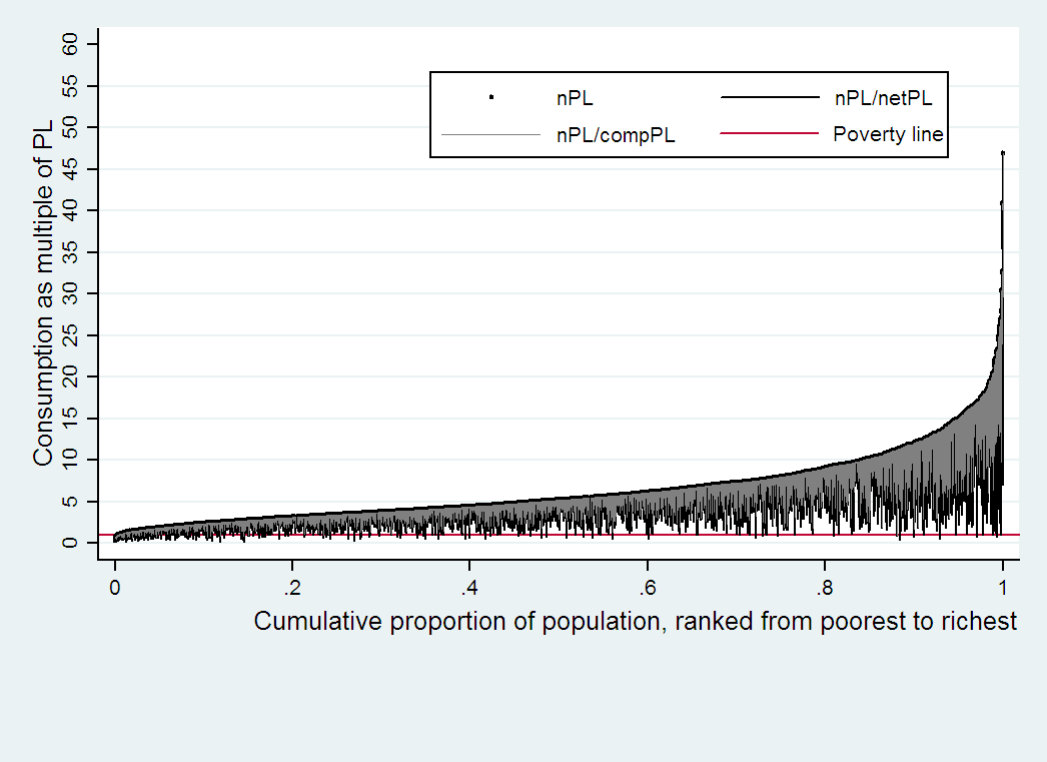
Effect of health payments and NCMS compensation on Pen’s parade of the household consumption distribution for hospital admission insured. Respondents’ economic status declined sharply and even crossed the poverty line when health payments are netted out. This illustrates that the health payments play a significant effect on household economic status for those who has been hospitalized. And insurance compensation helps alleviate the poverty status to some extent.

Pen’s parade of the hospitalized population also described the above changes (see Fig 2). Respondents’ economic status declined sharply and even crossed the poverty line when health payments are netted out, regardless of the initial economic status. Insurance compensation helps alleviate the poverty status to some extent.

## Discussion

Providing financial protection for insured is the main target of establishing medical insurance. A few studies have measured the impact on health payment-induced poverty or NCMS alleviating medical impoverishment. Gao and Zhou [22] evaluated the effectiveness of the NCMS in reducing health payment-induced poverty by comparing the NCMS with another experimental model called Rural Mutual Health Care (RMHC) in Zhen’an County, and indicated health payment made the poverty headcount of sample population decrease 20.35 percent, while NCMS reimbursement helped to decline 4.59 percent. Yan also used poverty headcount, poverty gap and mean positive poverty gap to evaluate medical impoverishment in Mei County [23]. For all NCMS insured, health payment increased the above indicator relatively 91.93 percent, 95.00 percent and 0.95 percent, respectively. For hospital admission insured, health payment increased those 532.91 percent, 3863.50 percent and 667.31 percent, respectively.

In this study, we assessed the effect of China’s NCMS on alleviating the health payment-induced poverty in Shaanxi Province, using commonly adopted poverty indexes. The health payment induced the poverty headcount of all the insured and inpatient to increase 127.84 percent and 1566.67 percent, the poverty gap increased 166.96 percent and 3222.54 percent, the mean positive poverty gap increased 167.14 percent and 3025.00 percent, respectively. The NCMS reimbursements helped the poverty headcount of all the insured and inpatient to decline 16.70 percent and 72.21 percent, the poverty gap declined 19.60 percent and 82.59 percent, the mean positive poverty gap declined 3.56 percent and 37.40 percent, respectively. Comparing the results with previous studies, it indicates that in Shaanxi Province, the effect of health payments on poverty was more severe, whilst the financial protection effect of the NCMS was stronger

As the result of National Health Accounts in 2013, total health expenditure increased faster (10.74%) than GDP (7.65%) [24]. Although the proportion of personal out-of-pocket in China’s total health expenditure declines every year (declines from 34.34% to 33.88% in 2013), health expense post even larger financial risks for the poor in rural China. So health payment could make even more household fall below the poverty line.

As NCMS keeps expanding its coverage since 2003, almost universal coverage has been achieved in rural residents by 2013[25]. Comparing the initiation of NCMS, financing standard, compensation rates and celling increased a lot. For example, total premium for each rural resident in Shaanxi Province were 50 Yuan in 2005 and 90 Yuan in 2008. As a consequence, financial protection effect of the NCMS became stronger. Along with economic growth, further research should investigate how to sustain the health insurance financing in rural China and meanwhile continue to improve the benefit package design of the NCMS scheme. Our study suggests a more comprehensive insurance benefit package design (enhance financing and compensation rate) could further improve the effectiveness of poverty alleviation.

The national poverty line used in this study was 2300 Yuan per year, announced by the NBS in 2012. This poverty line amounted to US$1 per day, and is lower than the absolute poverty line of US$1.25 per day that the World Bank determined in 2008[26]. Consequently, when comparing results in this study with international literature, the poverty headcount may be underestimated. However, the same poverty line was used to compare the poverty indexes before and after the household got reimbursement from NCMS. As we mainly focus on the relative difference, the conclusion of this study would be reliable no matter which poverty line was used.

There are some limitations in this study. Firstly, as mentioned in the Method section, owing to the data availability, the financial protection effect of health insurance on outpatient services was not considered. Consequently, the financial protection effect of the NCMS reported in this study might be underestimated. Secondly, since hospitalization expenditure and household consumption expenditure were both self-reported, there could be recall bias in the data. However, in the large-scale household survey, self-reported health service utilization and expenditure is widely adopted in the literature.

In summary, the NCMS could achieve the policy target of alleviating the health payment-induced poverty in Shaanxi Province.
